# Realisation of an Application Specific Multispectral Snapshot-Imaging System Based on Multi-Aperture-Technology and Multispectral Machine Learning Loops

**DOI:** 10.3390/s24247984

**Published:** 2024-12-14

**Authors:** Lennard Wunsch, Martin Hubold, Rico Nestler, Gunther Notni

**Affiliations:** 1 Group of Quality Assurance and Industrial Image Processing, Faculty of Mechanical Engineering, Technische Universität Ilmenau, Gustav-Kirchhoff-Platz 2, 98693 Ilmenau, Germany; gunther.notni@tu-ilmenau.de; 2 Fraunhofer Institute for Applied Optics and Precision Engineering IOF Jena, Albert-Einstein-Str. 7, 07745 Jena, Germany; martin.hubold@iof.fraunhofer.de; 3 Zentrum für Bild- und Signalverarbeitung e.V., Werner-von-Siemens-Str. 12, 98693 Ilmenau, Germany; rico.nestler@zbs-ilmenau.de

**Keywords:** multispectral imaging, multi-aperture camera, spectral processing, spectral sensor modeling, multispectral data analysis, feature space evaluation, machine learning, waste classification

## Abstract

Multispectral imaging (MSI) enables the acquisition of spatial and spectral image-based information in one process. Spectral scene information can be used to determine the characteristics of materials based on reflection or absorption and thus their material compositions. This work focuses on so-called multi aperture imaging, which enables a simultaneous capture (snapshot) of spectrally selective and spatially resolved scene information. There are some limiting factors for the spectral resolution when implementing this imaging principle, e.g., usable sensor resolutions and area, and required spatial scene resolution or optical complexity. Careful analysis is therefore needed for the specification of the multispectral system properties and its realisation. In this work we present a systematic approach for the application-related implementation of this kind of MSI. We focus on spectral system modeling, data analysis, and machine learning to build a universally usable multispectral loop to find the best sensor configuration. The approach presented is demonstrated and tested on the classification of waste, a typical application for multispectral imaging.

## 1. Introduction

In recent years, developments in the field of camera technology have led to systems capable of capturing scenes not only in spatial resolution, but also optical with spectral resolution. In combination with other imaging principles for non-optical scene features, our environment can even be captured and analysed multimodally. This additional information opens up new possibilities for scene analysis via new multimodal image recognition. This also requires the exploitation of newer ML techniques in order to recognise and use the sometimes hidden information and connections in the scene. Regardless of this, the provision of the most significant primary information possible in the camera data remains an important key to ensuring the efficient analysability of this valuable data source.

Pixel features from spectral reflection, being a material specific characteristic, enable a non-invasive, non-destructive, optical assessment of objects. The ability to detect a wide variety of spectral characteristics is an important source of information for differentiating between materials. Depending on the type and number of materials to be distinguished, a well-chosen set of spectral features can provide a subset of specific information to distinguish between materials.

Spectral imaging can be divided into hyperspectral and multispectral imaging. While hyperspectral imaging acquires hundreds of registered contiguous spectral bands, this type of recording leads to unnecessarily redundant spectral image information [1]. Meanwhile, multispectral imaging systems acquire only a subset of specific discrete bands of an object spectrum [1]. Hyperspectral images may contain more information; however, they also require high computational power, and also capture redundant information. Therefore, the usage of multispectral imaging systems can be useful for applications requiring only a small specific portion of the spectral range, since only a smaller, more relevant amount of information can be captured [2]. Furthermore, the signal quality of the individual channel responses can be significantly improved by the spectrally correlated broadband coverage of a spectral band.

Finding these application-specific spectral bands to distinguish different materials before completely designing the multispectral imaging system is necessary. Therefore, an extensive spectral analysis is performed beforehand. Capturing multispectral images can either be achieved sequentially by changing the spectral vicinity [3], or by using a snapshot approach with an spatially divided setup for spectral diversity [4].

One way of sequentially capturing multispectral images is the utilisation of a filter wheel camera system [5,6,7]. A different way of utilising a sequential imaging method is described by Bolton et al. [3]. The system uses a sequential activation of 13 different LEDs as illumination to image different bands within the visible and near-infrared spectral range. For image acquisition, a monochrome camera is used. Bolton et al. [3] present a proof of concept based on skin tissue monitoring.

Another way to capture multispectral images is by using a snapshot system [8,9,10,11]. In [4], we introduced a system using a tilted linear variable spectral filter and a micro-lens array in front of a common image sensor to distinguish between different continuous spectral bands within one single exposure. In this work, we consider a multi-aperture camera system which utilises distinct different spectral band-pass filters in a snapshot system.

Regardless of whether point sensors or imaging techniques are involved, there are various approaches to optimising the design of multispectral sensor systems for specific applications. In most cases, the aim of optimisation is to maximise the usable sensor information content from the spectral extracts to be generated, taking into account a specific application or general implementation efforts [12,13]. On the one hand, the literature attempts to derive optimal spectral system properties (e.g., in [13,14]) or to realise the best possible system behaviour by optimisation in a discrete parameter space from the given possibilities and known knowledge about the application [12], either pursuing a globally optimal result by brute force selection or, in the case of a large selection variety, by evolutionary [15], randomised methods driven by quality functions. In the first case, the challenge lies in the technological implementation of a spectrally optimised design proposal with subsequent necessary data processing (calibration of the sensor responses) in order to compensate for the effects of unavoidable spectral manufacturing tolerances in the realised integral sensor values [12]. In the latter approach, generally known data analysis methods (e.g., discriminant analyses [16] or algebraic decomposition) are used additionally at the sensor response level in order to generate features that are as independent and meaningful as possible for a given application class from a reliably realisable channel selection.

Machine learning (ML) based on such sensor responses enables a wide range of possible uses, including process prediction and optimisation [17,18], object classification [19,20], error detection [21,22], and determining the composition of materials [23,24]. For object classification, defect detection, and sorting, visual data such as images can be used. Therefore, with new image acquisition technologies, new possibilities for ML tasks emerge.

However, solving a ML task requires information. This information is preconditioned by the ML task itself. To avoid information redundancy, determining the best setup to capture a dataset according to the ML task requires an extensive preliminary analysis. In ML, this process is called feature selection.

The literature presents different approaches to spectral feature selection. In [2], multiple supervised and unsupervised methods are compared for spectral band selection, with information entropy and regression tree performing best. In [25], orthogonal total variation component analysis is used for feature extraction in hyperspectral images. By minimising the optimisation problem, the authors extract only significant features. In [26], unsupervised PCA-based methods are used for feature extraction. Since PCA transforms the feature space, this method can also be used for feature selection by considering the resulting contribution of features to the principle components. While these references focus on hyperspectral images, a more general overview of feature selection methods is given in [27,28]. Both present a generalisation of the feature selection process and evaluation methods. While [27] focuses on evaluation methods and applications, [28] concentrates on the methods themselves. In [28], feature selection methods are classified into filter-based, wrapper-based, and embedded methods, with filter methods not utilising a learning algorithm, wrapper methods evaluating selected feature subsets based on learning algorithm performance, and embedded methods selecting features during the learning process itself.

The modified mutual information presented in [29] is an example for filter based feature selection methods, using the mutual information itself as a measure of redundancy. A wrapper-based method is presented in [30]. The authors utilise an ensemble learning algorithm consisting of a decision tree and naive Bayes classifiers. In [31] a wrapper-based two-stage algorithm is applied to select features for the classification of polycythemia. After a local maximisation stage is used to select a subset of a fixed size, float maximisation is used to vary the selected subset in order to find a better subset. Later, the approach is also combined with a classification algorithm to improve its results further.

In the case of feature selection for the realisation of a multispectral imaging system, no labels are given by prior knowledge. Therefore, the problem at hand is an unsupervised problem, ruling supervised methods out.

The following questions must be answered in order to design a specific multispectral sensor, e.g., a multi-aperture sensor, in line with requirements:

1. What technology can be used to realise the spectrally selective channels in MSI? What variants are available and what spectral effects are associated with them?
2. How many spectrally selective channels are actually required; can an optimum compromise be found between optical-technological realisation effort and MSI sensor data usability?
3. How is the behaviour of the multispectral sensor described for objects of a selected application class?
4. Which are the best subsets of spectral channels with respect to the primary class separation of a given application class?
5. What is the best sensor configuration in the context of a machine learning task?
6. What is the necessary and possible number of usable channels for a sufficiently good multispectral classification?

The transfer of the proposed analysis concept to other multispectral imaging systems enables the sensible utilisation of existing spectral sensor and application data in order to improve suitability for specific applications. This primary image information with maximised information content for the recognition task makes downstream processing methods easier to implement with higher quality. In the case of ML-based evaluation with deep neural models, resource- and inference time-optimised model selection is made possible.

**Figure 1:**
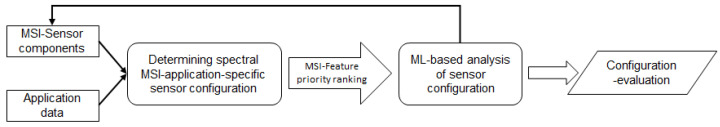
Proposed workflow and loop to find the best multispectral sensor configuration for a given application scenario.

Figure 1 shows the general workflow of the proposed method. The complete process is divided into two major processing steps. First the spectral-MSI-application-specific-sensor configuration is determined, before an extensive ML-based analysis of the proposed configurations is applied.

In the following chapters, the multispectral snapshot sensor is described (Section 2) and the used data are presented (Section 3). Afterwards, the proposed process is described in detail in Section 4, while the ML-based analysis of the determined sensor configurations is described in Section 5. In the end, the results are presented and raised questions answered (Section 6), before future works are considered in Section 7, and a conclusion is given in Section 8.

## 2. Multispectral Snapshot Sensor Based on a Multi-Aperture Camera

**Figure 2:**
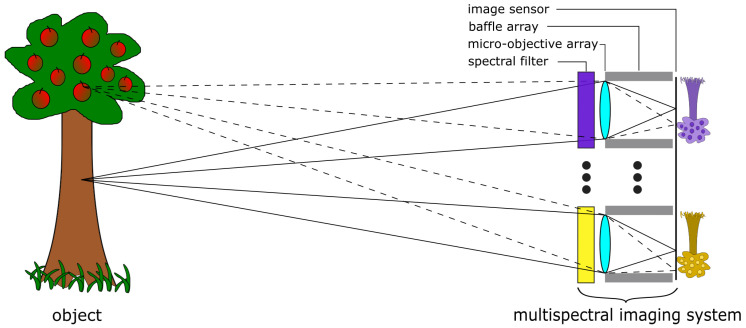
Scheme of multispectral multi-aperture camera principle.

The multispectral snapshot camera is based on a multi-aperture, micro-optical imaging system, in which each individual micro-optical imaging unit, hereinafter referred to as a channel, images the entire object distribution onto a common image sensor (see Figure 2). The object distance is several times larger than the focal length of each micro objective. In addition, each individual channel is equipped with a spectral filter element either near the systems’s aperture or in or near the image sensor plane, where customised image sensors with tiled filter arrays [32] or a customised cover glass made of pixelated spectral filters [33] are required. There are various spectral filter technologies, like linear-variable filters (LVF), band-pass filters (BP), or Fabry–Perót filters, that can be applied to the system. Furthermore, a baffle array is essential for the overall design, which serves as a field aperture to suppress spatial crosstalk between adjacent channels and false light outside the FoV. The physical size of the baffle structures defines the fill-factor, which represents the ratio of the usable tiled image areas to the total size of the image sensor size. This is how the entire spectral data cube is captured in a single shot without an additional bulky objective lens.

### 2.1. Filter Approaches and Image Sensor Sensitivities

Spectral band-pass filters on a single glass substrate with a varying passband along a single physical direction are referred to as linear variable filters (LVFs). It is beneficial to rotate this filter around the optical axis of the micro-objective array by a specific angle in order to achieve linear spectral sampling across all channels using a monolithic element (see [4]). The limited gradient of such a continuously varying filter necessitates the use of relatively large image sensor sizes, and the suppression of the blocking range is less effective than that of high-performance single-standard BP filters with steep edges. Such band-pass filters with an optical density (OD) greater than 4 are produced by a prominent optics manufacturer in substantial quantities. However, the BP filters with a narrow passband and broad blocking range over a wide wavelength range require a significant amount of dielectric interference-layer coatings, often comprising up to 100 or more individual layers. This results in total coating heights of 10–20 μm, dependent on the wavelength. The coating technologies that are available in-house were limited to a total layer thickness of approximately 5 μm. A combination of long and short-pass filters on both sides was calculated with relatively small pass bands and high transmission; however, the blocking range was completely insufficient. An alternative approach to the manufacture of narrow band filters is the use of metal interference filters (MIFs) [34,35]. The functionality is based on the Fabry–Perót principle, whereby a dielectric low-absorption spacer layer is embedded between two thin, partly transparent metal layers. In addition to the straightforward configuration and the minimal overall layer thickness, the key benefit is the extensive blocking range afforded by the metallic layers and the straightforward wavelength scaling of the passband, which is dependent on the spacer thickness.

**Figure 3:**
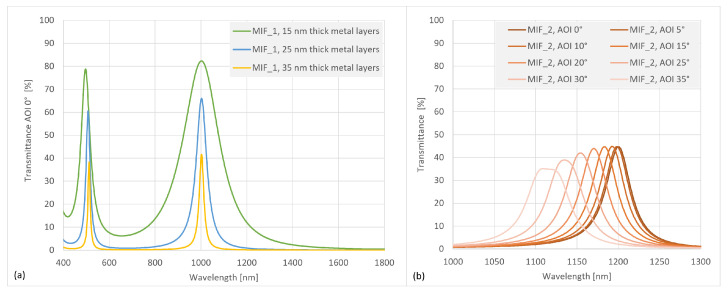
(**a**) Spectral transmittance profile of a MIF system at 1000 nm with variation of metal layer thickness. (**b**) Spectral transmittance of a MIF system at 1200 nm dependent on different AOI.

Figure 3a illustrates the calculated spectral transmission of the MIF under consideration, comprising a thin metal layer and a dielectric spacer layer, for a wavelength of 1000 nm. The maximum transmission in the passband is lower than that of purely dielectric band-pass filters, due to the absorption of the partly transparent metal layers. The transmission peak observed at approximately 500 nm is attributed to the second interference order and may be mitigated by incorporating an additional long-pass filter on the reverse side of the substrate. Moreover, the bandwidth of the MIF is dependent upon the thickness of the metal layer. It can be observed that an increase in the thickness of the metal layer results in a narrowing of the transmission peak and an improvement in blocking efficacy. However, the maximum transmission peak decreases rapidly. It is necessary to identify a compromise between the quality of the blocking and the height of the transmission in order to optimise the filters. Furthermore, Figure 3b illustrates the angle of incidence (AOI) behaviour of a MIF at 1200 nm. The transmission profile exhibits a pronounced broadening at large AOI, accompanied by a moderate decline in peak transmission. The maximum wavelength shift is observed to be approximately 30 nm at 20° AOI and approximately 80 nm at 35° AOI.

In addition to the spectral transmission profiles of the MIFs, the image sensor to be utilised in the multispectral multiaperture camera must be selected. Classical CCD or CMOS image sensors operate within a wavelength range of approximately 400 to 1000 nm, with pixel pitches down to approximately 1 μm and a range of sensor resolutions. Photodiodes comprising indium-gallium-arsenide (InGaAs) exhibit sensitivity within the short-wave infrared range, spanning approximately 900 to 1700 nm. By modifying the crystal structure, it is possible to alter the sensitivity range, extending it to higher wavelengths up to 2.2 μm [36]. In the event that a combination of visible and short-wave infrared wavelength ranges is desired, new image sensors have emerged that utilise a variety of technologies. For example, SWIR Vision Systems, Emberion, Imec, and STMicroelectronics utilise colloidal quantum dot (CQD) thin film photodiodes manufactured monotonically on silicon readout wafers, and Sony applies a combination of compound semiconductor InGaAs photodiodes and silicon readout circuits through a Cu–Cu connection, designated SenSWIR™. The latest CQDs have pixel pitches in the range from 20 down to 5 μm, identical to specifications of SenSWIR image sensors; however, ST Microelectronics have already demonstrated further downscaling to a 1.62 μm pixel pitch [37]. The image sensor properties of pixel pitch and sensor resolution and the resulting physical sensor size require special attention in the subsequent sensor tiling.

### 2.2. Micro-Objective Array, Object Scene and Baffle Structures

A micro-objective array consists of one or more stacked micro-lens arrays (MLA), depending on the complexity of the optical design. As previously stated, each individual channel of the micro-objective array within the multispectral MAC images the entire object field on a specific tile of the image sensor. Accordingly, the greater the number of spectral channels used, the lower the spatial resolution of the corresponding channel, and vice versa. Each individual channel generates an image circle. In order to create a rectangular array of image tiles with a high fill factor, the image circles must be confined by a rectangular baffle structure that serves as a field aperture in close proximity to the image sensor. The rectangular array of micro-lenses, with dimensions of m×n, permits only a discrete number of maximum channels, such as 4,6,8,9,10,12,14,15,16,18,20, and so forth, if each channel is assigned to a unique spectral wavelength range. The final tiled image ratio is dependent upon the format of the image sensor and the maximum number of channels.

The MLAs are fabricated as polymer-on-glass optics via a suitable mastering technology and a subsequent replication step on a wafer scale, which offers cost-effective elements for large quantities. The mastering process may be either a reflow of photoresist or ultra-precision manufacturing of a metal master. The aforementioned master structures are utilised for the fabrication of replication tools. In comparison to injection molding technologies, one- or double-sided MLAs exhibit precise buried black apertures. However, the achievable shape and sag height of the micro-lenses is restricted, which consequently limits the spatial resolution and f-number of each channel. These size and shape constraints were incorporated into the optical design process. Furthermore, the general object scene was defined, particularly the object field of view with a width of 20 cm due to the intended application (see Section 3) over a conveyor belt. The objective field of view of a single channel is dependent on the object distance. Assuming a constant object field, a reduction in object distance results in an increase in FoV, and vice versa. The resolution of objects is inversely proportional to the number of channels in an array. Moreover, the resolution of the object size increases with a reduction in the field of view (FoV) at a fixed object distance. The f-number *F/#* of each micro-objective is calculated in accordance to classical camera systems

(1) $$F/\#=\frac{f}{D_{EnP}},$$

where *f* represents the effective focal length and $D_{EnP}$ denotes the entrance pupil diameter of the objective lens. Reduction in the focal length and an increase in the aperture size are the two main techniques used to achieve a small *F/#*. However, both of these approaches result in aperture- and field-related aberrations, which must be corrected by the use of several large, thick, and complex lenses. Moreover, the maximum diameter of the largest lens in the system is constrained by the channel pitch of the micro-lenses, as they are not permitted to intersect. Ultimately, the aberrations must be maintained at a minimum to ensure sufficient contrast across the field in the final image, which is dependent on the properties of the image sensor.

Given that the application calls for a multispectral camera, it is necessary to correct chromatic aberrations over a wide wavelength range. The correction of chromatic aberration can be achieved through a number of well-established techniques. One such approach is the design of achromatic or apochromatic lenses using materials with differing Abbe numbers and focal lengths. Alternatively, a hybrid approach may be employed, utilising refractive and diffractive surfaces with opposite dispersion behaviours. However, both of these approaches result in increased system complexity and, consequently, higher costs. A cost-effective solution has been identified in the form of multi-aperture imaging, which uses lenses with adjustable radius of curvature (ROC) for different wavelength ranges. Each channel can be corrected specifically for the desired spectral band, as defined by the filters.

Due to the predominantly short focal lengths of the MLAs, which are only a few millimetres, an issue arises that does not exist in classical objective lenses, such as C-/F-Mount. This issue concerns the thickness of the cover glass and the distance to the actual focal plane array. In the event that the focal length of the micro-objective is less than the distance between the cover glass and the focal plane array, the image will inevitably lack sharpness. Moreover, the cover glass introduces aberrations into the optical system. It can be concluded that image sensors with these properties, such as TEC cooling, are not suitable. The baffle array serves two functions: it acts as a field stop and suppresses unwanted light in neighbouring channels. Therefore, the baffle walls must be thin and positioned close to the focal plane array in order to achieve a high fill factor. Consequently, it would be preferable to choose an image sensor with removable cover glass for the integration of customised micro-optics, which would result in a high fill factor baffle array and fewer aberrations.

### 2.3. General Dependencies and First Boundary Conditions

Initially, when the spectral filter is positioned in close proximity to the aperture, a spectral shift in the central transmission wavelength is observed, which is dependent solely on the angle of incidence and directly correlated with the field of view (FoV). However, this shift is not affected by the f-number. When non-telecentric imaging optics are used to achieve a high fill factor, the position of the spectral filter close to the focal plane results in an aperture and field-dependent behaviour of the spectral transmission profile. Moreover, the finite thickness of the substrates with MIFs on top introduces additional baffle structure issues for the suppression of out-of-FoV light and alignment issues during the assembly process. Additionally, stray light originating from the reflection of the MIF within the optical system must be considered. In the subsequent stages of the optical design process, the filter is positioned in close proximity to the aperture. Imaging a small field of view per channel reduces spectral dependencies, but only a higher f-number is achievable due to the limited micro-lens diameter size in the array. The fabrication of the baffle structures is also more challenging due to the necessity of thin baffle walls with a high aspect ratio.

The objective is to utilise the so-called butcher block method for the production of the spectral filter array. To this end, MIFs are coated with a low layer thickness gradient over a larger elongated substrate, which results in small variations in the filter characteristics, similar to those observed in a linear variable filter. A lateral resolving spectral measurement of the peak transmission on the substrate is utilised to assign, dice out, and join together individual pieces in a filter array. This method offers the advantage of omitting the lengthy spectral adaptation of narrow band filters on a specific pass wavelength. Additionally, a single coating run is sufficient to manufacture a multitude of spectral graded filters.

The Sony IMX990 image sensor [38] was selected for further consideration due to its small pixel pitch of 5 μm, its broad sensitive wavelength range (400–1700 nm), and the fact that the image sensor version features a removable cover glass. The image sensor has a 1/2″ format, with 1.34 megapixels at 1296(H)×1032(V) pixels. A 12-channel system would yield a resolution of approximately 0.11 MP/channel, while a 20-channel system would achieve only 0.067 MP/channel. Given our assumptions regarding object resolution of less than 1 mm/px for a 200 mm wide object field and the constraints on the minimum dimensions of diced spectral filters for assembly and handling, we limited the number of spectral filters to 12 for the MAC.

## 3. Spectral Characterisation of the Application Class

In recent years, there has been a growing awareness of the need for an environmentally friendly society in the face of climate change and increasing damage to natural resources. One way to reduce society’s environmental impact is to utilise construction waste resources efficiently. Therefore, the reuse of used products, known as recycling, is an environmentally friendly practice. By sorting the materials left behind after the demolition of buildings, some materials can be reused for new construction projects, while harmful materials such as asbestos can be disposed of properly. Depending on the use, this requires analysing and separating building material mixtures by type.

In this paper, we address the problem of automating the resource recycling of building material waste as an example application for the use of multispectral imaging as a primary data source for optical building material characteristics.

For the spectral characterisation of the application class (see Section 4, the so called App-set) we used 2366 point spectra of construction rubble data. The spectra were acquired between 380 nm and 1800 nm with a sampling rate of 5 nm using the Cary 5000 UV-VIS-NIR spectrometer by Agilent Technologies, Santa Clara, CA, United States and annotated in eight categories based on DIN 933-11 [39] at MFPA Weimar and spectrally preprocessed. Data pre-processing was of great importance here, as artifacts from the spectral measurement, e.g., measurement uncertainties and measurement-related discontinuities, were eliminated and redundant spectra were removed from the spectrum set. The automatic pre-processing of the large amounts of spectral data was carried out using the SpecWorks™1.3.0.0 - spectral prototyping tool (www.zbs-ilmenau.de/specworks, accessed on 1 February 2024). This tool was also used for the spectral sensor simulations in Section 4 and Section 5.

## 4. Method for Determining an Application-Related Multispectral System Configuration

The principle of application-related configuration of a multispectral sensor pursued here follows the criteria-driven selection of an amount of *S* from technologically given, realisable options *N*. In contrast to the determination of a parametrically spectral simulated and optimised configuration of a given sensor technology, the questions of the fundamental practical feasibility of the results are subsequently eliminated. In addition, there is no need for a fully parametric system model.

The primary data basis here is therefore a max *N*-multispectral sensor configuration $MSI^{MA,N}:MSI_{n}^{MA}\left(\lambda \right)$, which in this case consists of the combination of sensitivities of the sensor base material $S^{MA}\left(\lambda \right)$, the optical effect of filter layers to produce *n* spectrally selective properties $T_{n}^{MA}$, and optionally, an illumination source $I^{MA}\left(\lambda \right)$ for observed objects that do not emit radiation themselves. Furthermore, the annotated spectral characteristics of *L* objects Obj to be optically multispectrally detectable are required, which describe the radiation–object interaction of the spectral application data set App. In our specific application case, these are previously measured spectral remissions of different building material classes (see Section 3 above).

Using a linear model of the multispectral sensor, the measurable *n* channelwise sensor responses Resp can be integrally determined as shown in (2) and (3).

(2) $$Resp^{Obj,N}:Resp_{n}^{Obj}=\int_{\lambda }T_{n}^{MA}\left(\lambda \right)\cdot I_{MA}\left(\lambda \right)\cdot S_{MA}\left(\lambda \right)\cdot R^{Obj}\left(\lambda \right)\cdot d\lambda$$

(3) $$Resp_{n}^{Obj}=\int_{\lambda }MSI_{n}^{MA}\left(\lambda \right)\cdot R^{Obj}\left(\lambda \right)\cdot d\lambda .$$

In the chosen general approach, the spectral illumination characteristic can be considered both as a system component and as a component of the detected spectral stimulus. In either case, this also determines the channel selection. In order to derive a meaningful subset S≤N from a countable set of *N* possibilities, it is possible to test all existing options. However, due to the combinatorial diversity, this brute force strategy leads to too much effort, even for a manageable *N*.

For this reason, the proposed algorithm realises a step-by-step, quality factor-driven selection of a $MSI^{MA,S}$ by its simulated responses $Resp^{App,S}$ from a possible amount of *N*. The selection process starts with an initial configuration with only one selected sensor channel S=1. The most important ideas and parameters of the channel selection are defined and the process is described below.

In the case of non-spectrally characterised or characterisable MSI and application class, the following considerations can only be made on the basis of measured sensor responses $Resp^{App,N}$. However, this requires physically available systems in all variants.

The quality factor of a multispectral system configuration for a given set of application data App is determined primarily on the basis of the sensor responses $Resp^{App,N}$ or the ratios in the sensor feature space. In our case, it is a specially constructed kind of channel-wise (unimodal) statistic discriminance value cDisc based on [16] in the case of a set of C>2 object classes (see (4)). It describes the distinguishability of statistically moment-based described class ranges. Ideally, the discriminance value assumes the value infinity.

(4) $$cDisc_{n}=\frac{\sum_{c}^{C}\sum_{d\neq c}^{C}{\left(\mu Resp_{n}^{c}-\mu Resp_{n}^{d}\right)}^{2}}{\sum_{c}^{C}\sigma^{2}Resp_{n}^{c}>0}$$

Here, $\mu Resp_{n}^{c}$ is the mean sensor response of channel n∈N for the data class, *c* and $\sigma Resp_{n}^{c}$ characterises its standard distribution.

On this basis, a simple selection of sensor channels could be made from the largest sorted sequence of amount *N* of the channel-related discriminant values. This procedure realises a worst-case, i.e., without taking sensor feature correlations into account, selection by the best possible evaluation ratio in the sensor feature space.

Alternatively, a formal vectorial extension for a given multispectral configuration S>1 is possible by replacing the rational expression in Equation (4) with the so-called Mahalanobi distance Mah(c,d|S) value of class pairs.

(5) $$\begin{array}{cc} MDisc^{S} & =Mah(c\in C,d\in C|S)\\ Mah\propto {\left(\mu Resp^{c,S}-\mu Resp^{d,S}\right)}^{T} & \cdot Cov{\left(c,l\right)}^{-1}\cdot \left(\mu Resp^{c,S}-\mu Resp^{d,S}\right) \end{array}$$

Cov(c,l) is the covariance matrix, the scattering behaviour in a multidimensional feature space of dimension *S*. This type of multidimensional class spacing assessment is recommended, but this evaluation requires much computational effort.

In addition to the differentiability of class ranges in the sensor feature space, the overall correlation of the sensor responses is another optimisation criterion that determines the usability of a feature space.

Therefore, we add another criteria to control the selection of sensor channels. This is called the Mutual multispectral Channel Correlation Ratio (MmCCR). The multidimensional character of the feature space realised by the sensor and the application data set is taken into account here.

Useful for MmCCR are statistical or geometric scalar measures to formally evaluate the independence of vector sets. In our study, the normalised vector covariance is used as a representative of statistical measures or, alternatively, the vector cosine distance. Both focus on the ratio differences of the vector components to be evaluated and not on the absolute vector values and are therefore very robust in the application.

(6) $$S+k:\min_{l\in N,l\neq s}\sum_{s\in S}\left|MmCCR\left(\mu Resp^{App,l},\mu Resp^{App,s}\right)\right|$$

$$e.g.,\;geometric\;correlation\;rating\;MmCCR\left(A,B\right)=\sum_{c\in C}\frac{A_{c}\cdot B_{c}}{{∥A∥}_{2}\cdot {∥B∥}_{2}}$$

With the help of the evaluation in (6), it is possible to find the most suitable addition of a further channel to a given multispectral sensor configuration with S⩾1 channels from the remaining cDisc-sorted channel options. The evaluation in this paper uses the vector covariance. The cosine distance is particularly effective for larger amounts of data. Both correlation evaluations are sufficiently independent of global scaling of the sensor responses, e.g., due to different systematic modulations of the multispectral sensor responses as a result of exposure time or scene illumination.

In addition to the general feature space-based channel selection criteria described above, further spectral technological factors can be included in the procedure. In this way, in addition to the specific quality of the sensor data, properties of the technical realisation and the application implementation are also taken into account.

For example, channels that are overlapping, i.e., technologically redundant, in terms of their spectral characteristics can be excluded from a remaining selection set before each supplementation step. In addition, known primary tolerances of the spectral channel properties, e.g., of certain optical filter principles, can already be taken into account in the selection step. In this way, channel proposals can be evaluated in terms of their contribution to the system calibration effort in the real application.

From this point of view, the present implementation is specialised for the multi-aperture imaging system from Section 2. However, the method proposed can be applied to any type of multispectral sensor system whose behaviour can be described either spectrally or by measurements.

## 5. ML-Based Analysis of Sensor Configurations

Based on the feature-channel priority ranking and system specific criteria such as implementation effort, technical availability or quality standards, multiple subsets can occur. Each of these subsets represent a different possible sensor configuration. In the case of multispectral imaging systems. the cost of spectral filters can be a factor, as well as their availability. Moreover, a quality-driven criteria, such as a required minimum performance, in combination with technical aspects, like higher lateral transmission modes of spectral filters, can also result in multiple subsets. By either blocking or not blocking these transmission modes the performance can be influenced, since information are either present or absent. However, blocking also creates additional technical-optical efforts. To compare all subgroups based on ML performance, a machine learning driven data analysis is used. First, data are generated by applying Equation (2) for each subset, setting all optical and sensor terms accordingly to match the subset conditions, while using the fixed object reflectivity. This results in different datasets, again representing different possible sensor configurations.

Utilising these simulated sensor response data, each sensor configuration can be compared based on performance of a ML classification algorithm, using each subset, respectively, to train said algorithm. However, ML classification algorithms require a set of hyperparameters to define the training process, with each set of hyperparameters being unique in terms of data–classifier combination. Depending on the set of hyperparameters, the classifier’s performance can vary. Therefore, the next step in the ML-based analysis is the determination of the best sets of hyperparameters corresponding to each subset. Utilising a grid search approach, the accuracy of the classifier is maximised. Since this process results in many training steps, support vector machines (SVM) [40] were chosen due to their robustness and fast training times. It is still a very powerful, classic classification principle in the field of pattern recognition. To determine the best fitting kernel for further SVM investigation, both the polynomial kernel and radial bias function (RBF) kernel were used in preliminary investigations. While the best performance of the RBF kernel peaked at 49.53%±3.34%, the polynomial kernel peaked at a 64.62%±3.16% classification rate. Therefore, a polynomial kernel was used in further investigations. To compare the ranking algorithm to a benchmark, a third subset was created based on an equidistant distribution of filter within the considered spectral range. Since lateral features are used for this evaluation, other classification methods such as flat neural neural networks, decision trees, and so on could also be used instead of SVM. Therefore, the used classification algorithm is interchangeable.

To find the necessary number of usable spectral channels, a SVM is trained using the best set of hyperparameters, as determined before. Starting with all spectral bands, the SVM is trained sequentially, while after one training the last ranking feature is removed until only the highest-ranked feature remains. A five-fold cross-validation is used again. In case of saturation of performance after adding another channel, one of two possibilities arise. Either there is no new information included in the newly added data, or the information from the newly added data is irrelevant for class separation. In both cases, the channel does not contribute to a better class separation, and is therefore redundant. By finding this saturation, the minimum necessary number of channels for spectral information-based class separation is found. Combining all results allows one to determine the best application-specific realisation of the presented Multi-Aperture based Snapshot-Imaging System.

## 6. Results

In the following, the illumination spectra in Equation (2) was considered ideal $I_{MA}\left(\lambda \right)=1\left(\lambda \right)$. For data normalisation, we perform a multispectral *white balance* with the responses of an ideal object $R_{Obj}=1\left(\lambda \right)$.

Moreover, to put the shown mean accuracies and standard deviation into context, by using only simulated one pixel spectral information, a limited amount of features is used. No textual or form based features are used.

### 6.1. Best Subset of Filter in Terms of Class Separation

A selection of 52 metal interference filters with different peak wavelengths in the main mode was considered. Together with the spectral sensor properties and the illumination, the filters led to channel sensitivities, which will be the subject of the evaluation of the sensor behaviour. Due to the limitation of the spatial resolution by the usable multispectral resolution of the sensor to be realised, a subset of max. 12 filters was determined. The optimum filter selection was determined for the given application class described in Section 3 by the optimisation procedure described in Section 4.

**Figure 4:**
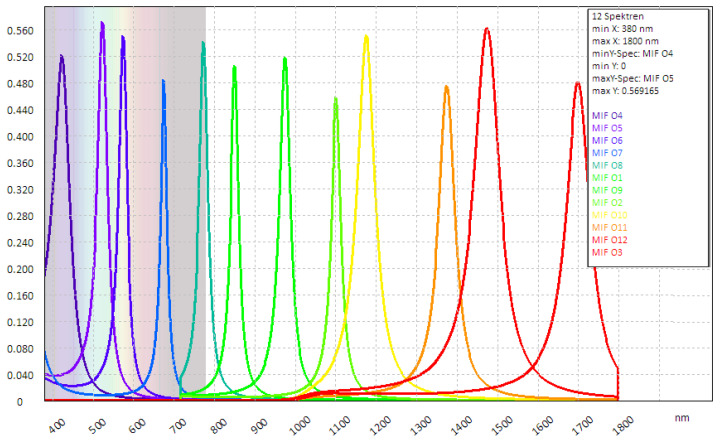
Application-specific optimised filter subset with priority sorting.

Figure 4 shows the selected filter subgroup. The filter order shown in the diagram on the right characterises the importance of the multispectral channel for the sensor decisions. The most important filter (corresponding sensor channel) comes first. The effect of this priority sorting in the selection process is particularly evident when fewer spectral channels are possible in favour of lateral sensor resolution.

**Figure 5:**
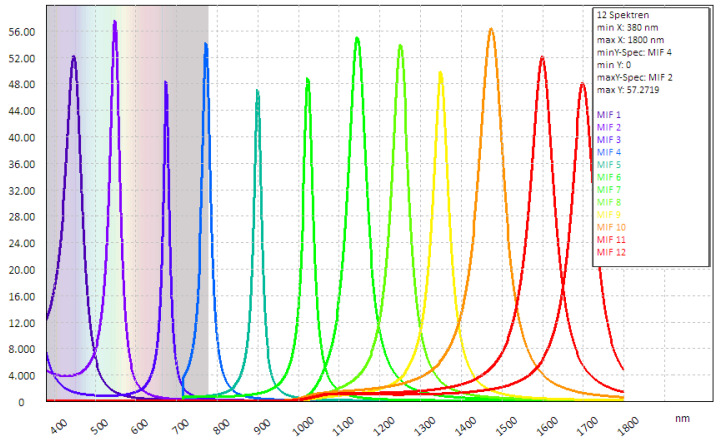
Arbitrary, spectrally equidistant filter selection without consideration of any specific application or priority sorting.

If the optimised filter subgroup is compared with an equidistant filter distribution, as shown in Figure 5, it shows that the optimisation leads to an automatic filter shift to where the spectra in the VIS and SWIR range differ significantly in terms of detection relevance. It was already known from the application that colour in particular plays a special role in recognition. This is confirmed by the optimisation (the four recommended channels).

### 6.2. Final MAC Parameters

**Figure 6:**
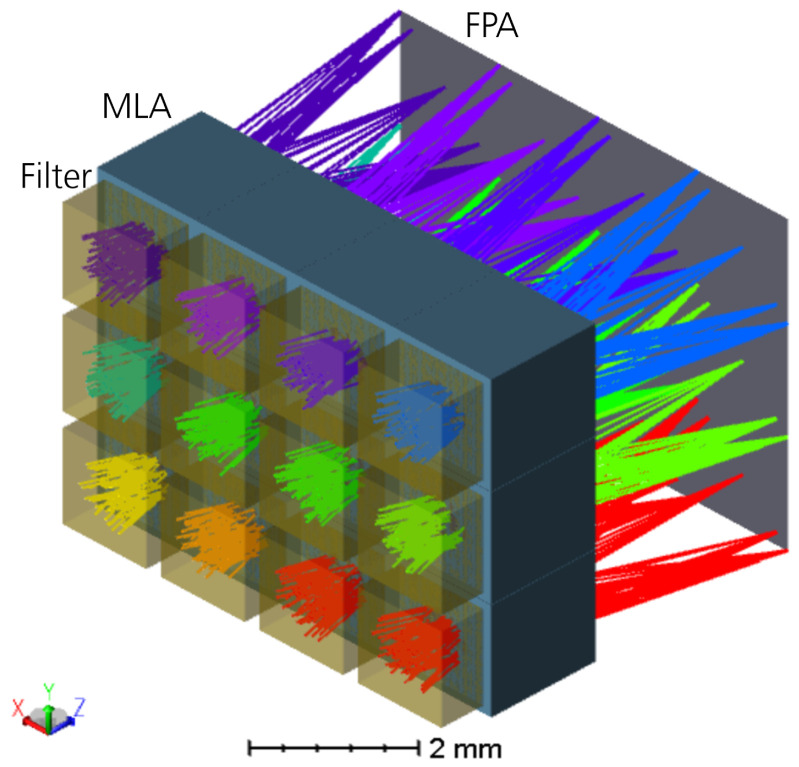
Isometric layout plot of the multispectral multi-aperture imaging system utilising a spectral filter array, an MLA, and an image sensor as FPA. The colours of the beams correspond to the colour representation from the spectral transmission profiles shown in Figure 4.

Given the overall system design is to be of low complexity and straightforward to integrate, the optical system (see Figure 6) comprises a one-sided plane-convex MLA with buried aperture diaphragms, and the filter array is bonded directly on the front side of the MLA substrate. The space between the spectral filter tiles will be filled with a black polymer in order to suppress possible stray light at the side faces of the filters. Furthermore, the cover glass of the image sensor is omitted.

The design parameters of the MLA are the aperture diameter, the thickness of the glass substrate, and the shape of the singlet lenses, with a variation in the ROC according to the optimised filter subset and the materials that are feasible for use. The selection of these parameters is based on the identification of a compromise for the system’s f-number over a reasonable field of view (FOV), which corresponds to the object distance and the spectral shift across the field. The contrast of the imaging system is characterised by the modulation transfer function (MTF) at a particular spatial frequency, which is used to evaluate its imaging performance. Subsequently, the MTF is optimised at a spatial frequency of $\nu_{0}=0.5\cdot \nu_{Ny}$, where $\nu_{Ny}$ is the Nyquist frequency, calculated by $\nu_{Ny}=1/\left(2p_{px}\right)$ using the pixel pitch of the image sensor [41]. The minimisation of the f-number for optimal light sensitivity, while maintaining a modulation transfer function MTF>0.5 at $\nu_{0}$ for each individual channel, whilst also maintaining a constant back focal length, represents the primary figure of merit of the optimisation process over the extended transmittance wavelength range from 400 nm to 1700 nm. The ROC and sag height of the lenslets of the MLA are constrained by boundary conditions imposed by the wafer-level manufacturing process, which utilises reflow-of-photoresist mastering. The final system parameters are presented in the following Table 1.

**Figure 7:**
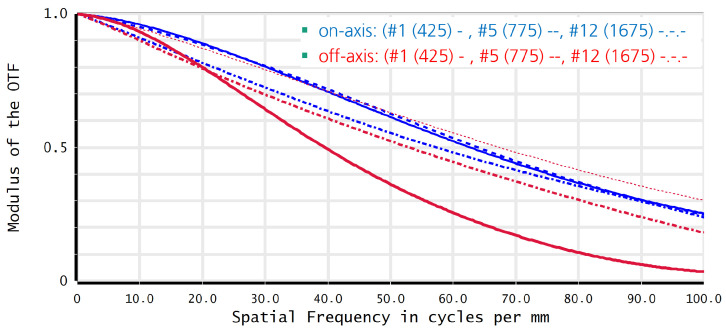
MTF plot of three different spectral channels (425 nm, 775 nm, 1675 nm) dependent on two field positions (blue: on-axis, red: off-axis).

Figure 7 illustrates the MTFs over spatial frequency for three distinct spectral channels: blue, red, and shortwave infrared wavelength range. The blue and red curves represent the on-axis and an off-axis field point, respectively. With the exception of a medium deviation of the off-axis blue spectral MTF, all MTFs exhibit a similar behaviour, thereby demonstrating the efficacy of the ROC adaptation for the correction of chromatic aberration, which in turn results in optimal contrasts in the image plane. A MTF of greater than 0.5 at 50 cy/mm (Nyquist-half frequency) is achieved for these channels. This is also the case for the other eight spectral channels above 550 nm, although this is not displayed in the figure for reasons of presentation simplicity.

### 6.3. Possible Configurations

**Figure 8:**
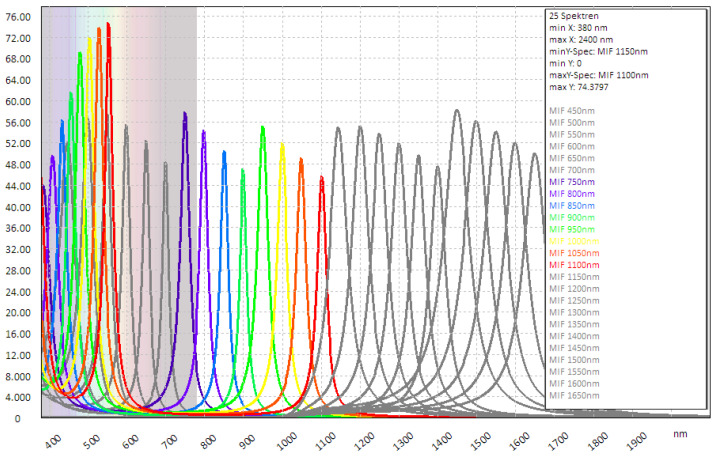
Some filter transmission profiles usable for MSI - camera configuration. Filters with higher order modes are shown in colour.

Based on the resulting significant bands, a set of spectral band-pass filters was chosen. However, the chosen band-pass filters had a higher order lateral mode. These lateral modes could either be ignored or blocked. This choice could result in two different viable filter configurations, with a third option being the benchmark with an equidistant filter distribution:

- 12 Channel MIF non-blocked;
- 12 Channel MIF blocked;
- Benchmark;

Non-blocked lateral modes allow transmission of the higher order modes in the VIS of electromagnetic waves, while blocked describes a configuration which is blocking these higher modes. In Figure 8, the higher order modes are displayed coloured. By comparing Figure 4 and Figure 8, the issues of these higher transmission modes becomes clear.

Having two possible sensor configurations results in two different datasets. Both are based on different sensor simulations according to Equation (2). Therefore, the next step is to determine which configuration performs better.

### 6.4. Comparing the Configuration

To determine the best configuration, the SVM performance for both resulting datasets (dataset description in Section 3) was compared. Utilising a grid search pattern, the best set of hyperparameters was found. Then, the best mean accuracy and standard deviation were compared after validating using a five-fold cross validation. Table 2 shows the accuracy of SVM classifiers trained on the blocked and non-blocked dataset, respectively, with different sets of hyperparameters.

Multiple things can be observed in these results. First, it is noticeable that the blocked as well as the non-blocked datasets’ performance exceeded the performance of an equidistant filter distribution (benchmark). Moreover, the best performance was achieved by blocking the higher lateral modes. Also, standard deviations achieved by blocking the VIS were lower than the corresponding non-blocked standard deviations. However, blocking does not always result in better performance. Therefore, the best performing configuration is also dependent on the chosen set of hyperparameters.

### 6.5. Minimum Required Number of Channels

While determining the required number of channels, the lowest ranking band was removed sequentially. Therefore, Table 3 shows the mean accuracies and standard deviations of both configurations with respect to the number of bands used for training.

An increase in the SVM’s performance when adding new bands can be observed. With an increase of 10 percentage points, the blocked dataset clearly outperforms the non-blocked version. In case of the nan values, training of the SVM took more than 14 h and was therefore not completed. While a general increase in the SVMs performance is noticeable, adding an additional band does not always have this effect. See the addition of band 5 for blocked and 4 for non-blocked. Blocking the VIS resulted in an overall performance peak; however, with a reduced number of bands, the non-blocked configuration outperformed the blocked one most of the times. Lastly, the blocked configuration achieved a lower standard deviation over the five-fold cross validation.

## 7. Discussion and Future Works

The evaluation presented shows a higher accuracy (see Table 2) for the SVM classifier used to evaluate the sensor information when using an application-optimised channel selection compared to an arbitrary equidistant selection. Furthermore, the higher accuracy seems to be independent of the hyperparameters. This shows a general increase in valuable information within the sensor data obtained by the MAC setup. The lower standard deviation also reveals that increasing the independence of the channel information by blocking the VIS increases the robustness of the trained SVMs.

The increase in the accuracy of the SVMs by adding more channels (spectral bands) can be seen in Table 3. This indicates additional information that can be added to the dataset by adding bands. Adding channels sometimes results in a decrease in performance. This is due to the introduction of redundant data. However, as accuracy generally increases, the overall impact of the additional redundant information remains small. Again, a lower standard deviation is seen when blocking the VIS, which emphasises the robustness of the trained SVMs.

In summary, it can be said that the approach propagated here and the methods used lead to multispectral sensors that are optimised both from the point of view of the actual sensor information and the technical-optical realisation effort. For the specific case of multispectral multi-aperture imaging, the existing conflict of objectives between spectral resolution and spatial resolution can be dealt with in an application-related manner.

Further investigations on this topic coiuld include the use of other classifiers and other classifier architectures and the transfer of the propagated method to other applications of multispectral image processing. The further development of the selection and evaluation loop procedure, e.g., by deeper integration of technological parameters or the determination and incorporation of information for spectral channel selection from a used classifier or prior knowledge, is also to be researched further in the future. In addition, a demonstrator system will be built in the future to obtain experimental data and to compare the results with our predictions about the usability of the sensor data obtained through the above simulations and evaluations.

## 8. Conclusions

Itn this paper, it was shown how a suitable configuration of a multispectral sensor can be determined by a multispectral ML loop on the basis of an application-related channel selection method and classifier-based feature space evaluation. Instead of spectral descriptions and spectral sensor system simulations, it is also possible to perform the channel selection and evaluation based on the calibration (measurement) data of the multispectral sensor for measured application objects.

In the specific case of the discussed multi-aperture multispectral sensor, the optimisation shown enables the number of spectral channels to be reduced from an available quantity to a number only required for the application. This alleviates the design conflict between the spatial and spectral resolution of the multi-aperture mutispectral sensor. The realisation costs for a multispectral sensor can thus be reduced. Therefore, the presented work enables an efficient use of multispectral imaging systems for recognition tasks.

Transferring the proposed analysis-concept to other multispectral imaging systems enables an efficient use of spectral sensor and application data in future applications. Being able to customise multispectral systems increases the benefit of industrial uses of multispectral imaging systems increases, due to an increase in performance associated with such customised systems.

## Figures and Tables

**Table 1:** System parameters of the designed multispectral MAC.

Property	Value
No. of channels	4 × 3
Array pitch (x/y)	1.65 mm/1.76 mm
Channel image resolution	300 × 320 px (including dead zone of baffle array)
Object sampling	0.6 mm/px
Fill-factor	86%
FoV per channel	40° (full-diagonal)
Nominal object distance	400 mm
F/#	3.9
Distortion	3.45% (barrel)

**Table 2:** Table showing the accuracy of SVM with different hyperparameters.

γ	*C*	Blocked in %	Non-Blocked in %	Benchmark in %
1	1	57.71±3.96	58.61±4.50	52.55±3.40
1	1.6	56.62±2.87	57.04±2.45	52.38±3.37
1	2	56.87±3.71	57.53±4.01	52.66±3.02
9.8	5.6	63.93±4.87	61.59±4.40	54.47±5.10
12.6	18	64.58±3.01	62.80±5.53	54.06±6.26
14	15.4	64.62±3.16	62.59±5.41	53.12±6.22

**Table 3:** This table shows the accuracy of optimised SVM with polynomial kernel on blocked and non-blocked datasets with γ=14 and C=15.4.

No. of Bands	Blocked Accuracy in %	Non-Blocked Accuracy in %
1	54.50±0.07	54.49±0.07
2	52.54±1.25	54.18±1.66
3	55.72±3.41	56.39±3.00
4	55.29±3.68	55.64±4.99
5	54.93±3.26	56.88±3.77
6	58.58±3.80	58.30±4.11
7	nan	59.87±4.99
8	58.89±4.60	61.38±5.12
9	60.79±3.69	61.28±5.73
10	64.32±2.13	nan
11	63.28±3.70	63.57±4.02
12	64.62±3.16	62.59±5.41

## Data Availability

The data and software that support the findings of this study are available from the corresponding author, [L.W.], upon request.

## Abbreviations

The following abbreviations are used in this manuscript:

AOI	Angle Of Incidence
CCD	Charge Coupled Device
CMOS	Complementary Metal Oxide Semiconductor
CQD	Colloidal Quantum Dot
MLA	Micro-Lens Arrays
FoV	Field of View
LVF	Linear Variable Filter
MAC	Multi-Aperture Camera
MIF	Metal Interference Filter
MmCCR	Mutual multispectral Channel Correlation Ratio
ML	Machine Learning
MSI	MultiSpectral Imaging
PCA	Principle Component Analysis
Poly	Polynomial
RBF	Radial Bias Function
SVM	Support Vector Machine
SWIR	Short Wave InfraRed
VIS	VISible Spectral Range
